# Mutagenicity of comfrey (*Symphytum Officinale*) in rat liver

**DOI:** 10.1038/sj.bjc.6602420

**Published:** 2005-02-22

**Authors:** N Mei, L Guo, P P Fu, R H Heflich, T Chen

**Affiliations:** 1 Division of Genetic and Reproductive Toxicology, National Center for Toxicological Research, US Food and Drug Administration, HFT-130, 3900 NCTR Road, Jefferson, AR 72079, USA; 2 Center for Hepatotoxicology, National Center for Toxicological Research, US Food and Drug Administration, Jefferson, AR 72079, USA; 3 Division of Biochemical Toxicology, National Center for Toxicological Research, US Food and Drug Administration, Jefferson, AR 72079, USA

**Keywords:** comfrey, transgenic rat, *cII* gene, pyrrolizidine alkaloid, tandem base substitution

## Abstract

Comfrey is a rat liver toxin and carcinogen that has been used as a vegetable and herbal remedy by humans. In order to evaluate the mechanisms underlying its carcinogenicity, we examined the mutagenicity of comfrey in the transgenic Big Blue rat model. Our results indicate that comfrey is mutagenic in rat liver and the types of mutations induced by comfrey suggest that its tumorigenicity results from the genotoxicity of pyrrolizidine alkaloids in the plant.

Comfrey (*Symphytum officinale*) is a tall perennial plant with large hairy leaves and small purple flowers (Winship, 1991; Betz *et al*, 1994). Comfrey is consumed by humans as a vegetable and a tea. It has been used as an herbal medicine for more than 2000 years to treat broken bones, tendon damage, ulcerations in the gastrointestinal tract, lung congestion, and joint inflammation, and to promote wound healing (Rode, 2002). Comfrey, however, is hepatotoxic in livestock and humans and carcinogenic in experimental animals. It induced hepatic veno-occlusive disease in humans (Ridker *et al*, 1985; Weston *et al*, 1987; Bach *et al*, 1989; Ridker and McDermott, 1989; Yeong *et al*, 1990) and hepatocellular adenomas and haemangioendothelial sarcomas in rat liver (Hirono *et al*, 1978). Although there are no epidemiological data regarding the carcinogenicity of comfrey, these adverse effects have raised questions of its potential carcinogenicity in humans. This concern led the US Food and Drug Administration to request voluntary removal of products containing comfrey from the market in 2001 (FDA, 2001). There are presently, however, no restrictions on the use of comfrey in many parts of the world.

There is little known about the mechanism of tumour induction by comfrey. Although induction of hepatic tumours has been associated with the pyrrolizidine alkaloids (PAs) that are present in comfrey, and PAs are genotoxic and carcinogenic by binding to liver DNA in humans and animals (Prakash *et al*, 1999; Fu *et al*, 2004), a comprehensive study of comfrey mutagenesis has not been conducted. This inspired us to investigate the mutagenicity of comfrey in rat liver, a target tissue for its carcinogenesis, by using a transgenic rat mutational model (Dycaico *et al*, 1994).

In this study, we evaluated the mutagenicity of comfrey in the liver *cII* gene of Big Blue rats. The treatment schedule was based on a previous study that evaluated the carcinogenicity of comfrey (Hirono *et al*, 1978). Comfrey roots were obtained from Camas Prairie Products (Trout Lake, WA, USA). Pyrrolizidine alkaloids in the comfrey roots were determined by mass spectral analysis. The PAs detected were similar to those reported previously (Betz *et al*, 1994), and included symphytine, 7-acetyllycopsamine, and 7-acetylintermedine as major components in near equal amounts; intermedine and lycopsamine were present in relatively smaller quantity (data not shown). To determine an appropriate dose for treatment, a preliminary experiment was conducted by feeding diets containing 2, 4, and 8% comfrey. Based on a minimum effect on weight gain, lack of overt toxicity to the liver, and a maximum effect on mutagenicity, a diet containing 2% comfrey root was chosen for the mutagenesis experiment (see Supplements 1 and 2). The comfrey roots were ground and then blended with basal diet powder (NIH-31 pellets, Purina Mills International, Brentwood, MO, USA) in a Hobart Mixer to make a 2% comfrey root diet. Groups of six 6-week-old male Big Blue rats (Taconic Laboratories, Germantown, NY, USA) were fed either a basal diet or the comfrey diet. The animals were killed after 12 weeks of treatment.

Mutant frequencies (MFs) were determined for the liver *cII* gene of the rats treated with comfrey (Table 1). The MF for rats fed comfrey was 146±15 × 10^−6^, which was significantly greater than the MF for control rats, 30±16 × 10^−6^ (*P*<0.001, ANOVA, Holm–Sidak test). In the previous study of the carcinogenic activity of comfrey (Hirono *et al*, 1978), rats receiving a diet containing 2% comfrey root had a 42% incidence of liver tumours, while no liver tumours were found in the control rats. This correspondence between mutation induction and tumour induction suggests that comfrey induces liver tumours through a genotoxic mechanism.

The mechanisms by which the carcinogenicity and mutagenicity of comfrey are produced are not fully understood. Although we encountered no overt signs of liver toxicity in our relatively short-term study, the liver histology of rats fed comfrey for prolonged periods is quite similar to that produced by some hepatotoxic PAs (Schoental, 1968; Hirono *et al*, 1976, 1977). Liver cell necrosis, haemorrhage, bile duct proliferation, and liver cirrhosis are frequently encountered even in rats from experimental groups that have no tumours. This suggests that the liver tumours in comfrey-treated rats might be induced by the PAs present in comfrey. Indeed, comfrey contains up to nine PAs (Stickel and Seitz, 2000; Kim *et al*, 2001; Schaneberg *et al*, 2004), at least two of which, symphytine and lasiocarpine, are carcinogenic when administered as pure compounds (Svoboda and Reddy, 1974; Hirono *et al*, 1979).

Recently, we investigated the mutagenicity of riddelliine, a representative genotoxic PA, in Big Blue rat liver. The most common mutation induced by riddelliine was G:C → T:A transversion; however, an unusually high frequency of tandem base substitution was also found (Mei *et al*, 2004). Since it has been suggested that all PAs produce the same types of DNA adducts (Fu *et al*, 2004), we hypothesised that if the PAs in comfrey were responsible for its mutagenicity, the mutational spectrum of comfrey should be similar to that induced by riddelliine. Therefore, we sequenced 211 mutants from comfrey-treated rats and 63 mutants from control rats. A total of 200 and 46 independent mutations were identified from the treated and control animals, respectively. The types of mutations detected are summarised in Table 2 and compared with *cII* mutations isolated from the livers of riddelliine-treated rats. Statistical evaluation of these spectra (Adams and Skopek, 1987) indicates that the spectrum from comfrey-fed rats is significantly different from the control (*P*<0.001), while there is no significant difference between the spectra induced by comfrey-treated and riddelliine-treated rats (*P*>0.05). G:C → T:A transversion (42%) was the major type of mutation in comfrey-fed rats, whereas G:C → A:T transition (43%) was the predominant mutation in the controls. In addition, an unusually high frequency of tandem base substitutions (17%) was observed among the mutations from comfrey-fed rats. Tandem base substitution has been suggested as a mutational signature for the genetic damage of PAs (Mei *et al*, 2004). Therefore, these mutational data support the hypothesis that the mutations induced by comfrey in rat liver are due to PAs in the comfrey.

In conclusion, treatment of transgenic Big Blue rats with comfrey induced mutations in the liver *cII* gene. This result suggests that comfrey induces liver tumours by a genotoxic mechanism. The mutational spectrum from comfrey-treated rats suggests that PAs in the plant are responsible for mutation induction and tumour initiation in rat liver.

## Figures and Tables

**Table 1:** Liver *cII* mutant frequencies in comfrey-treated and control transgenic Big Blue rats^a^

**Group**	**Total plaques screened (× 10^3^)**	**Mutant plaques**	**Mutant frequency (× 10^−6^)**	**Mean±s.d. (*n*=6)**
Control	528	15	28	30±16 × 10^−6^
	310	13	42	
	587	8	14	
	481	8	17	
	544	30	55	
	339	8	24	
				
Comfrey	254	34	134	146±15 × 10^−6^^b^
	285	41	144	
	298	43	144	
	288	50	174	
	215	32	149	
	225	30	133	

a Methods for performing the *cII* mutagenicity assay were described previously (Mei *et al*, 2004).

b Significantly higher than the control group (*P*<0.001; ANOVA, Holm–Sidak test).

**Table 2:** Summary of independent mutations in the liver *cII* gene from comfrey-treated, riddelliine-treated, and control Big Blue rats^a^

	**Control**		**Comfrey^b^**		**Riddelliine^b^^,^^c^**	
**Type of mutation**	**Number**	**%**	**Number**	**%**	**Number**	**%**
G:C → C:G	5	11	11	6	4	5
G:C → A:T	20	43	24	12	22	26
G:C → T:A	9	20	83	42	29	35
A:T → T:A	1	2	5	2	4	5
A:T → C:G	3	7	7	3	5	6
A:T → G:C	1	2	9	4	4	5
Frameshift	7	15	26	13	8	10
Complex	0	0	2	1	0	0
Tandem base substitution	0	0	33	17	7	8
Total mutants screened	46	100	200	100	83	100

a The mutants were sequenced using the Methods and Materials described previously (Mei *et al*, 2004).

b Spectra for comfrey- and riddelliine-treated rats are significantly different from the controls [*P*<0.001; Adams and Skopek test (Adams and Skopek, 1987)]; there is no significant difference between the spectra for comfrey and riddelliine (*P*>0.05).

c Riddelliine data are from literature (Mei *et al*, 2004).
