# Supplementary material for: Mutagenicity of comfrey (Symphytum Officinale) in rat liver
Source: Br J Cancer. 2005 Feb 22;92(5):873–5. doi: 10.1038/sj.bjc.6602420 (PMC2361893; doi:10.1038/sj.bjc.6602420)
Supplement: Supplements 1 and 2 [file 92-6602420x1.doc]

**Supplement 1.** The relative mean body weight curve for Big Blue rats fed with a diet containing 0, 2, 4 and 8% comfrey root for 12 weeks in a preliminary study. The rat relative body weight was calculated as a ratio of the body weight during the experiment to the body weight at 6 weeks of age when the feeding of comfrey started. The data represent the means for groups of 3 rats. (♦), vehicle control group; (■), 2% comfrey; (▲), 4% comfrey; (●), 8% comfrey.

**Supplement 2.** The liver *cII* mutant frequencies for Big Blue rats fed with a diet containing 0, 2, 4 and 8% comfrey root, respectively, for 12 weeks in a preliminary study. The data represent the means for groups of 3 rats.
